# Ethics approval in applications for open-access clinical trial data: An analysis of researcher statements to clinicalstudydatarequest.com

**DOI:** 10.1371/journal.pone.0184491

**Published:** 2017-09-08

**Authors:** Derek So, Bartha M. Knoppers

**Affiliations:** Centre of Genomics and Policy, Department of Human Genetics, McGill University, Montreal, Quebec, Canada; University of Oxford, UNITED KINGDOM

## Abstract

Although there are a number of online platforms for patient-level clinical trial data sharing from industry sponsors, they are not very harmonized regarding the role of local ethics approval in the research proposal review process. The first and largest of these platforms is ClinicalStudyDataRequest.com (CSDR), which includes over three thousand trials from thirteen sponsors including GlaxoSmithKline, Novartis, Roche, Sanofi, and Bayer. CSDR asks applicants to state whether they have received ethics approval for their research proposal, but in most cases does not require that they submit evidence of approval. However, the website does require that applicants without ethical approval state the reason it was not required. In order to examine the perspectives of researchers on this topic, we coded every response to that question received by CSDR between June 2014 and February 2017. Of 111 applicants who stated they were exempt from ethics approval, 63% mentioned de-identification, 57% mentioned the use of existing data, 33% referred to local or jurisdictional regulations, and 20% referred to the approvals obtained by the original study. We conclude by examining the experience of CSDR within the broader context of the access mechanisms and policies currently being used by other data sharing platforms, and discuss how our findings might be used to help clinical trial data providers design clear and informative access documents.

## Introduction

Recent years have seen a dramatic increase in the sharing of patient-level clinical trial data by the global pharmaceutical industry. There are a number of widely-agreed benefits from this movement. Data sharing honors the contributions of the original clinical trial participants; allows researchers to avoid the costs and risks of conducting a redundant study; ensures that data from non-replicable studies are available to researchers; facilitates meta-analysis of data from different studies; allows study results and regulator decisions to be independently verified; counteracts reporting bias and other barriers to data interpretation; helps patients, doctors and regulators make more evidence-based judgments; provides data for teaching purposes; contributes to the development of new statistical methods and analysis tools, such as machine learning systems; strengthens collaborative efforts between data users; displays gaps in existing research; reveals new trends, hypotheses, and priorities for future studies; helps to identify earlier endpoints for future trials; and builds trust in clinical research so as to encourage future participation [1–7].

Patient-level data sharing also raises a number of potential challenges, including: time constraints; difficulties performing quality assurance and standardization across platforms; the need to respect the privacy and informed consent of participants; misinterpretation of results; the abuse of data by business competitors; the need for appropriate recognition and credit sharing among researchers; increased burdens for regulatory agencies; potential consequences for regulatory review and patenting; and other legal obstacles [3,5,8–11]. It also presents new costs for trial sponsors, including those of “establishing a team of clinical and statistical experts to evaluate data requests, forming a legal team to draft and negotiate agreements regarding data use, monitoring of data requesters to ensure compliance with data-use conditions, and hiring of technical staff to set up databases and facilitate the use of data sets” [4,8]. These may be especially problematic for data sharing initiatives from lower income settings [7]. Yet despite these concerns, there is a strong prima facie case for the responsible sharing of clinical trial data [12].

In accordance with these principles, a number of journals, nonprofits, funders and regulators have adopted policies which mandate the sharing of anonymized patient-level data. In this context, anonymization has been defined by the Global Alliance for Genomics and Health (GA4GH) as “The irreversible delinking of identifying information from associated data” so that “it can no longer be used to identify a Data Donor by using all the means likely reasonably to be used by person or entity” [13].

*Nature*, *Science*, *BMJ* and *PLOS* journals, among others, require data availability as a precondition of publication [14–16]. While PLOS itself has always required authors to make their data available to interested researchers, it implemented a policy in March 2014 that mandates authors make data “fully available without restriction” immediately upon publication, while still taking any steps necessary to preserve participants’ privacy such as “de-identification, blocking portions of the database, or license agreements” [16]. JAMA has also expressed support for raw clinical trial data sharing in the interest of study replication, using “database deidentification and other legal restrictions” to reduce risks to participants [10]. In 2016, the International Committee of Medical Journal Editors (ICMJE) issued a proposal to require the sharing of de-identified patient-level data from clinical trials within six months of publication [2]; however, the same Committee announced in 2017 that, while new data sharing statements would be implemented, sharing itself would not become a general requirement for publication [17]. The *BMJ*, *Nature*, the *New England Journal of Medicine*, and *PLOS* have also agreed to the WHO’s 2015 consensus statement on timely data sharing during public health emergencies, initially convened in response to the Ebola crisis [18].

A number of medical research funders have also issued policies for patient-level data sharing. In 2010, a group of seventeen funders led by the Wellcome Trust and the Hewlett Foundation issued a broad statement of purpose on their commitments to increase data sharing [19]. One of these organizations, the Bill and Melinda Gates Foundation, notably announced in January 2015 that all data underlying results produced with its funding would have to be deposited immediately in an open and accessible data repository [20]. Moreover, data sharing from federally-funded research is mandated by the US Office of Science and Technology Policy [21] and the National Institutes of Health requires that applicants receiving $500,000 or more per year submit a data sharing plan if possible [22].

European drug regulators have also called for the development of data sharing standards [5]. While the European Medicines Agency (EMA) has plans to share patient-level data from the regulatory approval process, within the personal data protection requirements of the EU, this plan has yet to be implemented; at present, the EMA only makes anonymized clinical reports available to researchers [23,24]. The FDA proposed a similar policy in 2013, although this was not followed up on due to opposition from stakeholders like the Pharmaceutical Research and Manufactuers of America (PhRMA). In their official comment on the proposal, PhRMA pointed out that, in collaboration with the European Federation of Pharmaceutical Industries and Associations (EFPIA), they had already adopted a set of “Principles for Responsible Clinical Trial Data Sharing” set to be implemented in January 2014 [11,25,26].

In accordance with these goals, a significant number of major pharmaceutical companies have made data from their completed clinical trials available to researchers. The first and largest platform for controlled access to this type of data is ClinicalStudyDataRequest.com (CSDR), which was launched in May 2013. CSDR currently manages access to 3049 trials from thirteen sponsors: Astellas, Bayer, Boehringer Ingelheim, Daiichi-Sankyo, Eisai, GSK, Lilly, Novartis, Roche, Sanofi, Takeda, UCB and ViiV. All of these have committed to the EFPIA-PhRMA principles, with the exception of ViiV (which is itself a creation of signatories GSK and Pfizer) [27, 28].

Each CSDR sponsor has an information page which sets forth a number of limitations on data access. All 13 companies explicitly exclude data that would be difficult to anonymize, usually giving the examples of rare disease studies, single-center studies, and other trials with small enrollment. Only Sanofi states that “genetic/genomic data” poses similar limits on anonymization. Every sponsor except Lilly also states that their ability to share data is limited by the terms of their patients’ informed consent. 10 of them specify that informed consent may limit new research to the same medicines or diseases as the initial study aimed to investigate; of these, all except Astellas and Eisai also state that they have begun asking clinical trial participants to consent to data use for broader indications (mostly starting in 2014, when the EFPIA-PhRMA principles took effect). Three companies, Boeringer Ingelheim, Roche, and UCB, mandate that data users publish their results in academic journals. However, none of the 13 sponsors discuss local ethics approval among their criteria for access [29].

The CSDR website states elsewhere that “Researchers are responsible for gaining any other approvals that are required for the research (for example, from Ethics Committees, Institutional Review Boards, relevant research institutions or funding bodies)” [30]. Similarly, CSDR’s data sharing agreement requires that the “lead Researcher will obtain any regulatory or ethics approvals necessary to conduct the Research Proposal” [31]. However, only researchers requesting Bayer’s trials are required to provide CSDR with a copy of their approval. This additional rigor is in keeping with the high standards common in German bioethical practices, although no specific reasons for differences between sponsor requirements are discussed on the website.

Since March 2015, research proposals submitted to CSDR have been examined by an Independent Review Panel (IRP) run by the Wellcome Trust [31, 32]. The IRP’s 2014 Charter notes under “Independent Review Panel Membership” that its panel members have expertise in a range of areas including “ethics”. The Charter also includes a list of “assessment criteria” for research proposals. Although one of these criteria asks “whether the proposal has potential to produce information that will enable identification of individual research participants”, the submitted proposal’s actual ethics approval status is not mentioned explicitly in the Charter [33].

Nevertheless, in June 2014, CSDR added a question to its clinical data request form which asks “Does your proposed research require Ethics Committee or Institutional Review Board (IRB) approval?” Applicants who select No are asked to “provide further details” in explanation [34]. CSDR does not provide any guidance as to when approval might be “required” or about the type of “further details” which might be relevant to the IRP’s consideration of a project without ethics approval. Thus, it is assumed that applicants have sufficient regulatory knowledge to be aware of whether projects using existing clinical trial data ought to have ethics approval in their local context and to answer the query on the data request form. Approvals from “relevant research institutions or funding bodies” are not included in the question.

CSDR’s terms of service state that the information collected from applicants may be used for “research purposes” and that CSDR retains “full use of all information acquired through this site that is not in personally identifiable form.” Given the present lack of information on researchers’ knowledge and use of controlled access systems, we decided to examine CSDR applicants’ responses to the question on ethics exemption in order to determine which reasons were most commonly used to justify the lack of local ethics approval for research projects using patient-level clinical trial data.

## Methods

Between June 23, 2014 and February 14, 2017, CSDR received a total of 172 research proposals, including 61 which stated that their projects required ethics approval and 111 which stated they did not. At the time of data collection, 62 of those 111 had been approved without conditions, 28 were approved with conditions, 13 were declined with advice to resubmit, 3 were withdrawn, 3 were still under review, and 2 were declined outright. Of the submissions with ethics approval, 43 were approved, 9 were approved with conditions, 4 were declined with advice to resubmit, 2 were withdrawn, 2 were still under review, and 1 was deemed invalid. Thus, projects with and without ethics approval were approved at roughly similar rates.

CSDR has only rejected 14 applications for not meeting requirements, mostly because “the research proposal is not seeking access to anonymised patient level data”, although one was rejected because “the research proposal did not meet the sponsor policies for informed consent”. CSDR also displays the reasons for which enquiries about non-listed studies were declined. These are listed on a sponsor-by-sponsor basis, although there is presently no data available from Bayer. Numerous requests have been turned down due to an inability to properly anonymize the relevant data, and Lilly has declined two “due to the informed consent not providing information explaining the additional use of data to patients in the study”. However, none of these requests have been rejected due to explicit concerns about the ethics of the research proposal [35].

While information on applicant jurisdictions was not included in the data used for this article, the CSDR website lists this information for approved studies. These include projects from Canada, the United States, the United Kingdom, Spain, France, the Netherlands, Belgium, Germany, Austria, Italy, Switzerland, Denmark, Sweden, Jordan, India, China, Taiwan, South Korea, Japan, and Australia. Both academic and corporate researchers were represented. The number of requests also varied significantly by sponsor, with 91 requests approved for CSDR’s earliest sponsor, GSK, and 0 requests so far approved for data from Daiichi-Sankyo [35].

Raw data including researchers’ responses to the ethics exemption question were received from CSDR in the form of Excel tables. The responses were each read and coded with an emergent keyword based on why they said ethics approval was not required. These were not considered mutually exclusive, and responses were coded with as many keywords as seemed appropriate. New categories were added to the list of possible codes over the course of the coding process. Each response was then double-checked against any new codes added this way. After discussing the initial codes, the authors chose to combine a number of results into categories encompassing similar justifications. Several new codes were identified when updated data was received from CSDR on February 14, 2017, upon which this process was repeated. The full text of the applicant statements and their coding results are available in S1 File, with any potentially-identifying information such as application number and institution redacted.

## Results

The codes identified from the CSDR data and the number of researchers who mentioned each of them are listed in Table 1. Most applicants gave multiple reasons for their project’s ethics exemption, and the average statement to CSDR mentioned 2.1 distinct codes.

**Table 1:** Coding results from ethics exemption statements.

Reason for Exemption	Mentions (out of 111)
De-identified / anonymized data	64
Re-analysis of existing data / post-hoc analysis	60
Jurisdiction	16
Consulted IRB	15
Covered by other approval	14
Already published / public	13
University policy	12
Patients already consented	11
No interaction with patients	11
Other	6
Within scope of original research question	5
No new recruitment	3
No risk	3
Conducted following Helsinki Declaration	2
None	1

A table indicating the number of applicants who mentioned each of the listed reasons as justification for not requiring ethics approval for their research proposals to ClinicalStudyDatarequest.com

Broadly speaking, there were four general themes into which these codes could be aggregated. The most common of these was the lack of potential effects upon patients (including the codes “de-identified”, “no interaction”, “no recruitment”, and “no risk”), which appeared in 63.1% of statements. For instance, one researcher wrote “This study is just to re-analyze the available data,which needn't [sic] to do any further sampling, data collecting, etc. And all subjects will be anonymous, which will make sure that all information will be protected from these subjects.” An American applicant to CSDR also cited the US Common Rule, which exempts the use of information from which “subjects cannot be identified, directly or through identifiers linked to the subjects” [36]. Meanwhile, the EU has legally recognized that data is not “personal” if the techniques used to irreversibly remove or de-link it from individuals’ identifiers and attributes means that there is an extremely small risk of their being re-identified under “the means likely reasonably to be used” by any party [37].

The second-most common theme, and the only other one which appeared in over half of the responses, included references to the use of existing data (including the codes “reanalysis” and “already published”). One or more of these codes appeared in 56.8% of statements. For instance, one researcher wrote “The aim of this study is to perform a metaanalysis of published data to produce a correlation… It is only based on published data and therefore no ethics committee is needed.” Existing data (and even more so published data) here refers to datasets from completed and already approved studies, whether their outcomes were successful or not. It should be noted that, although the research proposals received by CSDR include a variety of statistical methods including predictive models, meta-analyses and survival analyses, 84% of applications to CSDR are from researchers working on “a new study and publication” rather than just confirming the results of the original trial [32]. Independent verification is one of the chief benefits of data sharing, but only a limited number of researchers have pursued this so far.

The third most common theme represented appeals to ethical, regulatory or legal authorities (including “IRB”, “university”, “jurisdiction” and “Helsinki Declaration”), which appeared in 33.3% of statements. For instance, one researcher wrote “The Ethical Committee of [University Name] recently stated that this kind of research on de-identified data is not encompassed by the Law of Ethical Trials, but also that they had no objections to the planned study”. The individual code of “consulted IRB” was the fourth most common overall, indicating that many researchers were willing to take steps to avoid contravening the terms of data access even when it was not necessary to directly submit an ethics approval form. This may also indicate that many researchers experienced a level of uncertainty regarding the process.

The final major theme involved the approvals obtained by the original study (including “patients already consented”, “other approval”, and “scope of original research”), which appeared in 19.8% of statements. This most frequently referred to the original approval of the clinical trial being requested, but involved a number of different specific explanations. For instance, one researcher wrote “The primary end point that we will be using is the same primary end point as used in the vaccine efficacy trials–whether an individual developed the vaccine-preventable disease. Our use of the data aligns with informed consent because the objective of vaccine efficacy trials is to better inform vaccine policy decision making, which is also the goal of our research. Our research will increase the value of the information obtained by the vaccine efficacy trial.” References to informed consent could encompass the original patients’ specific consent to further analysis following the completion of that trial or a more general consent to the use of their data following anonymization. Other respondents appeared to be citing their own previous approvals for related projects, or even the CSDR database’s own approval to store and share patient data [38]. One application stated that ethical approval was not required in their jurisdiction but that the researchers had received an approval for data handling, indicating a somewhat broader take on the scope of the question.

## Discussion

Applicants to ClinicalStudyDataRequest.com provided a number of different, yet often overlapping, explanations as to why their projects were not subject to ethics approval. Their research proposals almost always invoked one or more justifications from the four primary categories described above, with anonymization or de-identification as the most common themes. However, the statements studied in this article varied greatly in both length and content, ranging from just a few words to full paragraphs with citations. This may indicate that researchers interested in viewing clinical trial data are not used to providing this sort of information. As such, it is possible that this data more closely reflects the assumptions among international researchers than an accurate snapshot of the pertinent regulations in each of their jurisdictions. In a previous article, CSDR suggested that “a lack of knowledge about the system” might be one explanation for the limited number of applications received as of 2016 [32]. If so, it may be also reasonable to expect this constraint to be reflected in applicants’ statements on ethics approval.

A second factor which may be contributing to this ambiguity over ethics expectations is the lack of clear guidance in the existing standards for data sharing. The EFPIA-PhRMA “Principles for Responsible Clinical Trial Data Sharing” state that, starting January 1, 2014,“each company will establish a scientific review board that will include scientists and/or healthcare professionals” who will determine whether requests meet criteria “regarding the qualifications of the requestor and the legitimacy of the research purpose” [26]. There is no explicit mention of ethical review or the involvement of ethics experts, although a number of signatories have provided further detail on their own websites (Table 2).

**Table 2:** Ethics approval instructions by EFPIA-PhRMA principle signatories.

Obtain ethics approval if needed but don’t submit documentation; otherwise, explain why not needed	CSDR sponsors other than Bayer (Astellas, Boehringer Ingelheim, Daiichi-Sankyo, Eisei, GSK, Lilly, Novartis, Roche, Sanofi, Takeda, UCB, ViiV Healthcare) [34]; Genzyme (as part of Sanofi); Shire [39]; Baxalta (as part of Shire)
Obtain ethics approval if needed but don’t submit documentation	AbbVie [40]. Bristol-Myers Squibb (via SOAR) [41]; Celgene [42]; Leo Pharma [43]; Lundbeck [44]; Otsuka [45]; Servier [46]
Obtain ethics approval if needed and submit documentation	AstraZeneca [47]; Bayer [34]; EMDSerono [48]; Novo Nordisk [49]; Pfizer [50]
No information on ethics approval	Almirall [51]; Amgen [52]; Biogen [53]; Grunenthal [54]; Johnson & Johnson (via YODA) [55]; Menarini [56]; Merck [57]; Orion [58]
System under development	Bial [59]; Chiesi [60]
No information on patient-level clinical trial data sharing	Esteve [61], Ipsen [62], The Medicines Company [63], Vifor Pharma [64]

A table indicating the ethics approval guidelines for researchers submitting data access requests to companies that have signed the EFPIA-PhRMA “Principles for Responsible Clinical Trial Data Sharing”.

In 2015, the US Institute of Medicine published a report titled “Sharing Clinical Trial Data: Maximizing Benefits, Minimizing Risk”. The report recommends the development of transparent norms including the use of data sharing agreements, privacy protections, and IRPs with lay representatives. While the review of applicant qualifications and research proposals is discussed, there are no recommendations regarding ethics review [4].

Also in 2015, the UK’s Medical Research Council released a set of “Good Practice Principles for Sharing Individual Participant Data From Publicly Funded Clinical Trials” to be employed by Clinical Trials Units with controlled access policies. This document, which is endorsed by the National Institute for Health Research and the Wellcome Trust, actually uses CSDR as one of its case studies. It emphasizes the importance of developing a clear and transparent data request policy but does not discuss the review of applicants’ ethics approval status [65].

The European Federation of Statisticians in the Pharmaceutical Industry (EFSPI) has a primer for academic researchers accessing patient-level clinical trial data which provides more guidance. It notes that researchers should check if their institution or country requires ethics approval, that the responsibility for getting such approval rests with the researchers, that some holders of data may ask for proof of approval, and that medical ethicists may be included on the review board, although it does not discuss whether ethics approval will be examined by the reviewers [66].

Lastly, the Multi-Regional Clinical Trials Center of Brigham and Women’s Hospital and Harvard is currently developing a nonprofit initiative “to enable stakeholders to comply with PhRMA, EFPIA, EU and IOM guidelines” regarding clinical trial data sharing [67], and is also involved in the creation of an independent platform for clinical trial data sharing called Vivli [68].

There are also a number of more general standards for data sharing from biobanks, which are not specific to patient-level clinical trial data sharing but could also apply to these platforms. These vary greatly in terms of the information they provide: The International Society for Biological and Environmental Repositories (ISBER) states that repositories should establish sharing policies consistent with ethical standards; that Data Transfer Agreements may include requirements for ethics approval; that proposals should include ethics approval documentation if required; that reviews should take “ethical considerations” into account; and that identifying information should be removed from the data unless the investigator has ethics approval to view it [69]. The OECD’s 2009 “Guidelines on Human Biobanks and Genetic Research Databases” state that access agreements may include requirements to obtain ethics approval when needed and that requests should include an “ethically appropriate research plan” [70].

GA4GH has a ‘Framework for Responsible Sharing of Genomic and Health-Related Data”, which is addressed to many stakeholders including ethics committees, but does not include further information on ethics approval [71]. It is, however, spearheading efforts to improve the consistency of ethics review processes for multi-jurisdictional research projects, including the release of its GA4GH’s 2017 “Ethics Review Recognition Policy”. This policy notes that “ethics principles, policies or norms that are mandatory in one jurisdiction should be respected in regard to this jurisdiction” while those which are not mandatory should managed formally or through the application of “international ethical principles” [72].

In a similar vein, many authors have pointed out the lack of harmonization among community standards for patient-level data sharing [7, 8]. Although there are a number of existing standards for research using controlled access clinical trial data and for health data more generally, some of which discuss ethics approval, there is rarely guidance on when approval might be required or what the platform’s IRP ought to expect from researchers. More specifically, there is a lack of discussion regarding the relevance of de-identification, prior publication, local regulations or previous ethics approvals to the review, meaning that most standards do nothing to address the most common justifications cited by CSDR applicants.

Similarly, the current landscape of controlled access platforms represents a considerable patchwork with respect to ethics approval (Table 2). Among signatories to the EFPIA-PhRMA principles, only Bristol-Myers Squibb, Otsuka and Pfizer explicitly include bioethical expertise in their IRPs. However, none of the three explicitly review submissions’ ethics as part of their charters. Novo Nordisk does review ethics, although it is one of the many companies without an ethicist officially listed on its IRP. Leo, EMD Serono and Novo Nordisk also require that researchers follow any conditions or instructions provided by their local IRB. Finally, Pfizer is the only company which requires the submission of annual proof of ethics renewal. Trial sponsors rarely present this information in a prominent fashion, and it is often scattered across multiple web pages and downloadable Word documents. (It should be noted that significantly fewer applications have been received by these other controlled access sites than by CSDR: of those that list metrics on their public websites, Pfizer has approved 35, Merck has approved 26, Amgen has approved 23, AbbVie has approved 15 requests, and Bristol-Myers Squibb and NovoNordisk have both approved 4. Grunenthal has received 0 requests) [40, 41, 49, 50, 52, 54, 57].

Data requests involving the AstraZeneca group are handled through a platform called PharmaCM, which is run by the company TrialScope on behalf of sponsors seeking to ensure their compliance with new data disclosure legislation [47, 73]. There are also a number of public and academic platforms for online clinical trial data sharing, including but not limited to BioLINCC, Project DataSphere, YODA, SOAR, CDAS and VISTA.

The Biologic Specimen and Data Repositories Information Coordinating Center (BioLINCC) provides access to both data and specimens from the National Heart Lung and Blood Institute. Its access requirements are the most stringent of any platform reviewed in this article. BioLINCC requires that researchers have their plan reviewed by a local IRB operating under OHRP-approved Assurance and the DHHS regulations laid out in 45 CFR Part 46, meaning in practice that the data is restricted to US-based applicants. It also requires that the IRB either approve the study or find it exempt from review; that evidence of review and/or approval be included with the request “where appropriate”; and restricts some research materials to applicants agreeing to abide by “IRB approval and/or complaints with other limitations”. Its own review process covers “Ethical considerations including consistency with the terms of the informed consent and compliance with human subjects and HIPAA regulations” [74].

Project DataSphere was developed by the CEO Roundtable on Cancer as a platform for phase III cancer clinical trials. Its current sponsors include Amgen, AstraZeneca, Bayer, Celgene, Lilly, Johnson & Johnson, Merck, Pfizer and Sanofi, among many others. The Project DataSphere user agreement holds researchers responsible for acquiring ethics review if required by law or institutional policy, but does not ask applicants to provide information on their approval [75].

The Yale University Open Data Access project (YODA) provides clinical trial data from Johnson & Johnson, as well as medical technology companies Medtronic and SI-BONE. It does not mention ethics approval on its website. While its data use agreements state that the researcher must have “all necessary authorizations, licenses, consents, and approvals”, this appears to be in the context of legal authority rather than IRB review [55]. Supporting Open Access for Researchers (SOAR) is a project run by the Duke Clinical Research Institute which provides access to clinical trial data from Bristol-Myers Squibb. Its website does not mention ethical approval [76]. Nor do the websites of the National Cancer Institute’s Cancer Data Access System (CDAS) or the Virtual International Stroke Trials Archive (VISTA) [77, 78].

As was the case for international standards, there is little agreement between different data providers regarding the need for ethics approval or its role in the review process. Nor is this information provided in a generally clear, prominent, or comprehensive manner.

It is true that any effort to provide researchers with information on local ethics approval is subject to a major limitation: controlled access requests come from many different institutions and jurisdictions, each of which may have unique requirements for researchers using patient-level data. Given that CSDR has previously approved applications from twenty different countries and from both academic and nonacademic researchers, it would be impossible to apply one standard to all possible applicants. Nor would it be practical for data providers to spend time researching the precise requirements applicable to each researcher interested in viewing the data, especially given that many of the relevant documents are in languages other than English. Regardless of whether the platform’s model requests ethics documentation or not, there does not seem to be an alternative to the existing method used by CSDR and other platforms which asks researchers to state in good faith whether they are exempt from ethics approval. However, the lack of guidance about what this question entails may limit the usefulness of their responses.

## Conclusion

Best practices for clinical trial data sharing should ensure that interested researchers are provided with all the relevant guidelines needed both to understand their obligations and to submit the necessary information to the IRP. Accordingly, numerous data sharing standards have recognized the need for clarity regarding data access requirements and procedures [4, 70, 71]. At this early stage in the data sharing movement, it is likely that the full ethical implications of the new research model are not presently apparent. Yet given the effort made by data providers to protect participant identities and honor their informed consent, it is also evident that ethics has an important role to play in clinical trial data sharing.

Unfortunately, none of the existing standards for this research model address the question of when local ethics approval is needed among applicants. This is reflected in the different approaches adopted by major pharmaceutical companies: although most ask the applicant to obtain ethics approval if needed, their websites differ as to whether documentation should be provided or not, whether the applicant is required to explain any exemption from review, whether the IRP will include ethical expertise, or whether the ethics of the applications will be factored into their assessment of the proposal. About a third of the sponsors examined did not mention ethics approval at all, and what information they do provide is often buried in legal agreements rather than featured as a part of the submission process. None of the companies sharing clinical trial data provide instruction as to what uses of data might qualify for exemption.

The main difficulty in providing this sort of guidance is that, given the broad international nature of this model of data sharing, legal requirements and other practices regarding ethics review may differ significantly from country to country and applicant to applicant. Specific statements on which sort of studies should seek ethics approval might be misleading or even false for many researchers. Nevertheless, it could be helpful for data providers to include more general comments on the uses of data which could be relevant to the approval process.

In order to explore researchers’ own stances on the need for ethics approval, we drew on ClinicalStudyDataRequest.com’s experience as the largest clinical trial data sharing platform by reading the ethics exemption statements provided by applicants. While there was some evidence that the applicants found their responsibilities unclear, the most frequently given reasons for exemption, by a significant margin, were the use of de-identified or anonymized data and the researchers’ intent only to re-analyze existing data. These general categories could be addressed in the research proposal submission process, such as by suggesting that academic researchers check their institution’s policies on the use of these types of data from human subjects. Platforms can also try to be clear about the role which local ethics approval plays in their IRP’s review process.

Patient-level data sharing from clinical trials has been a fairly recent development in the transparency movement. Indeed, a number of the companies examined in this article are still in the process of figuring out how to meet their obligations as signatories of the EFPIA-PhRMA principles. There is still great potential for the development of more harmonized approaches to clinical trial data sharing, and we suggest that this task should also include consideration of the place of ethics approval in this new research model.

To our knowledge, this is the first article to report on ethics approval among researchers applying for access to clinical trial data. As the use of such platforms becomes more widespread and application rates rise, further analysis of stakeholder needs will be necessary. We hope the line of inquiry opened by these results can ultimately assist data sharing initiatives in developing clear and helpful guides, standards and/or access documents which also accord the necessary importance to the protection of clinical trial participants.

## Supporting information

S1 File Raw CSDR data with coding. An Excel file containing the text of the researcher statements on ethics approval, along with the codes assigned to each entry.
(XLSX)
